# Advancing Equity in STEM: The Impact Assessment Design Has on Who Succeeds in Undergraduate Introductory Chemistry

**DOI:** 10.1021/jacsau.2c00221

**Published:** 2022-07-20

**Authors:** Vanessa R. Ralph, Leah J. Scharlott, Adam G. L. Schafer, Megan Y. Deshaye, Nicole M. Becker, Ryan L. Stowe

**Affiliations:** † Teaching Engagement Program (Office of the Provost) and Department of Chemistry and Biochemistry, University of Oregon, 1585 E 13th Avenue, Eugene, Oregon 97403, United States; ‡ Department of Chemistry, University of Iowa, 305 Chemistry Building, Iowa City, Iowa 52242, United States; § Department of Chemistry, University of Wisconsin—Madison, 1101 University Avenue, Madison, Wisconsin 53706, United States

**Keywords:** equity, STEM, assessment design, mechanistic reasoning, logistic regression

## Abstract

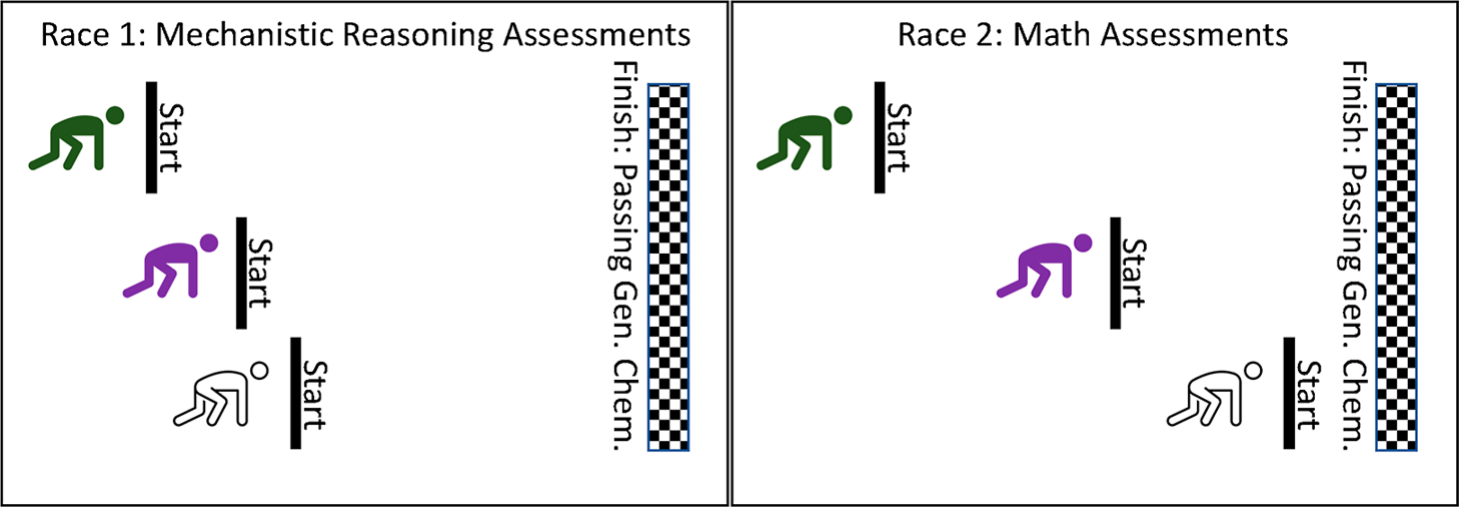

What we as scientists and educators assess has a tremendous impact on whom we authorize to participate in science careers. Unfortunately, in critical gateway chemistry courses, assessments commonly emphasize and reward recall of disaggregated facts or performance of (often mathematical) skills. Such an emphasis marginalizes students based on their access to pre-college math preparation and misrepresents the intellectual work of chemistry. Here, we explore whether assessing intellectual work more authentic to the practice of chemistry (i.e., mechanistic reasoning) might support more equitable achievement. Mechanistic reasoning involves explaining a phenomenon in terms of interactions between lower scale entities (e.g., atoms and molecules). We collected 352 assessment tasks administered in college-level introductory chemistry courses across two universities. What was required for success on these tasks was rote math skills (165), mechanistic reasoning (36), neither (126), or both (25). Logistic regression models predict that the intellectual work emphasized on in an assessment could impact whether 15–20% of the cohort passes or fails. Whom does assessment emphasis impact most? Predicted pass rates for those often categorized as “at-risk” could be 67 or 93%, depending on whether their success was defined by rote calculation or mechanistic reasoning. Therefore, assessment transformation could provide a path toward advancing the relevance of our courses and educational equity.

## Introduction

### Goals of Science Education

In this study, we examine how the decisions we make as instructors regarding what we emphasize on assessments in science courses impact the scientific community. Science is central to our modern world and fosters our ability to make informed decisions and understand relevant phenomena.^1,2^ However, science achievement and the emphasis on science in primary school curricula are declining in several countries.^3^ In the United States, downward trends in science achievement were identified following the early 2000s alongside persistent inequities in student outcomes and the diversity of their teachers.^4^ Concerns that K–16 science education in the United States is not adequately supporting learners have motivated many calls for “high-quality science for all students.”^5,6^

″High-quality science” was framed by the US National Academies as equitable, “hands-on” (or “active”), and centered on purposefully integrating scientific knowledge and practices (e.g., asking questions, analyzing, and interpreting data) to make sense of phenomena and design solutions to problems.^6^ Acting as a critical form of thinking for working scientists, mechanistic reasoning involves explaining “how the particular components of a system give rise to its behavior” through the interactions of components at least one scalar level below a phenomenon of interest.^7,8^ Figuring out how interactions between lower scale entities cause an observation of interest is central to a host of scientific and science-adjacent careers (e.g., chemist, medical doctor, and ecologist). Accordingly, curricular ecosystems that support mechanistic reasoning would be expected to effectively prepare future scientists and scientifically literate citizens. Thus, “high-quality science” courses should be structured to support learners in constructing and critiquing causal mechanisms for observable events.

There has been movement toward centering instruction and assessment on causal explanations of phenomena at the K–12 level.^6,9,10^ However, emphasis on mechanistic reasoning is far from the norm at the undergraduate level.^11,12^ Consider, for example, the following hypothetical lesson on the topic of solutions:

*Students are told the difference between solution, solvent, and solute and quizzed on these definitions via clicker questions. Then, they are tasked with representing dissolution of NaCl in water via a chemical equation. Finally, the class calculates the molarity of several solutions, given grams of solute and milliliters of solvent. Homework given after the lesson requires students to undertake several analogous molarity calculations*.

The design of this lesson signals that recall of (seemingly) random facts and skills is the goal of chemistry courses, not constructing plausible mechanisms for phenomena of interest to the class.^13,14^ Indeed, students in this vignette are never asked to explain how salt dissolves in water or why this can cause temperature changes. As we will see, the sorts of intellectual work emphasized in class and on assessments have a critical impact on both the quality of science education and whether it serves all students.

### How do the Goals of Science Education Affect the Community?

Most STEM majors and pre-health tracks require general chemistry. Unfortunately, general chemistry is one of the earliest science courses undergraduates encounter with high attrition rates (∼50% in some cases).^15,16^ Thus, these gateway courses have a lasting impact on who moves toward a STEM-centered career.

Some have taken to the use of linear modeling to predict which students are least statistically likely to succeed or be supported by a given educational system. Evidence of the correlation between students’ pre-college math test scores and grades in college-level chemistry courses is prevalent in research literature and spans multiple decades.^17−27^ The Scholastic Aptitude Test (SAT) and the American College Test (ACT) are popular college entrance exams in the United States and thus often used as the “math test” for these studies. Correlations between introductory chemistry outcomes and math test scores range from 0.42 to 0.63 for the SAT^21−24,28,29^ and 0.36 to 0.76 for the ACT.^20,30^ This suggests that differences in students’ access to pre-college mathematics preparation can account for up to 58% of the variance observed in chemistry tests. Indeed, students with lower scores on pre-college math tests than their peers disproportionately withdraw or receive D’s and F’s.^17−23,25,31−33^

If access to pre-college mathematics preparation were randomly distributed across the student population, student-level remediation may be a helpful approach. However, evidence suggests access to pre-college mathematics preparation is not equitably distributed, resulting in inequities in course pass rates observed across social constructs (e.g., gender, race, and ethnicity), disfavoring students marginalized and minoritized by the educational system.^30,34−38^ Therefore, we sought to examine system-level reforms that could reduce the penalty paid by students with limited access to pre-college mathematics preparation while also improving the quality of the science education students receive. In particular, we considered the role of assessments in science education in determining who succeeds and who does not.

### How Do Assessments Communicate the Goals of Science Education?

Research suggests that what we emphasize and reward on assessments plays a decisive role in messaging the knowledge products and processes that are valuable to students.^12,39−41^ How we assess students’ use of knowledge impacts their motivations, perceptions of self, the way they study, and what they judge is essential to learn.^31,42^ Furthermore, assessments send implicit messages to students about the nature of science. If we primarily assess decontextualized skills and factual recall, then students are likely to infer that the purposeless execution of algorithms is the point of science.^12,43,44^ Despite this evidence, tasks that emphasize recall and skills remain common,^45,46^ particularly in undergraduate chemistry courses,^44−47^ where it has been observed that 50–79% of the points awarded on high-stake assessments required students to perform a calculation.^12^

Perhaps students’ pre-college math test performances are highly predictive of their “success” in traditional chemistry courses^17−27^ because math skills are a major emphasis on assessments used to define “success”. This is extremely problematic, as overemphasis on disaggregated mathematical skills on chemistry tests acts to marginalize students with inequitable access to pre-college mathematics preparation. This chain of inferences suggests that the norms on science assessments may not be “high-quality” or aligned with the intellectual work of scientists. Importantly, evidence also indicates that such approaches to assessing science may not be “for all”.

Interestingly, there is prior work to suggest that improving the quality of assessment in science education could help to redress systems of inequity. For example, Lin and colleagues provide evidence that students who excel “conceptually” (rather than algorithmically) were often from minoritized student groups.^48^ Why might students who are minoritized by the system be so successful at “conceptual” tasks? Some insights may be gleaned from the work of Clark and Sieder, who reported the role of curiosity in the unique development of Black and Latinx students’ analytical thinking, critical consciousness, and involvement in activism.^49^ Clark and Seider’s findings suggest that Black, Indigenous, and Latinx students excel in learning environments that define academic success as nuanced reasoning and not rote execution or recall. As the construction of causal mechanisms is very often nuanced, it is plausible that Black, Indigenous, and Latinx students are uniquely well equipped for this sort of work. These studies subvert narratives about the deficits of “at-risk” students, offering an emphasis on their assets and revealing the limitations and strategies for improving the educational systems we participate in to prepare students for careers in science.

In summary, we hypothesize that the emphasis on rote math skills and the inequities observed in science course outcomes are related and may persist, in part, because we have not yet established new societal norms for the teaching, learning, and assessing of chemistry and, more generally, science. If we were to assess use of knowledge more reflective of scientific practice and removed from the rote, algorithmic strategies presumably emphasized in students’ pre-college preparations, we may support more equitable achievement. More generally, paying attention to the impact of assessment emphasis on who succeeds may provide one avenue to advancing equity in college-level science courses.

Therefore, in this study, we ask:

(1) How often mechanistic reasoning was emphasized on the assessments given to students in large-enrollment introductory chemistry courses, and
(2) How what was emphasized in these assessments influenced who was likely to succeed.

## Materials and Methods

### Experimental Design

#### Objectives

We sought to examine (1) how often mechanistic reasoning is emphasized in chemistry courses and (2) how differences in assessment emphasis may impact which students pass this large-enrollment, gateway STEM course.

#### Settings

As instructional practice (i.e., how students are taught) is often emphasized in education reform over course content (i.e., what students are taught),^50^ we opted for a cross-institutional sample collected at learning environments undergoing department-wide reforms in instructional practice and curriculum. Assessments were collected from each learning environment during the Fall of 2019 (General Chemistry 1) and Spring of 2019 (General Chemistry 2). Any references to “pre-college math test scores” in these settings refer to students’ registered math test scores on the ACT. All data collected at either setting were in accordance with the institution’s Internal Review Board.

Chemistry courses in the Practice Reform were structured according to the textbook *Chemistry: The Molecular Science*,^51^ have been observed to dedicate a substantive amount of class time (35%) to student-centered instructional practices (i.e., active learning),^50^ and had prerequisites for enrollment that included a “suitable” (but unspecified) math placement score or the completion of a college-level mathematics course.^52^ Forty-nine percent of a student’s grade in the first-semester Practice course consisted of their outcomes on four exams (three midterms, worth 10% each, and a final exam worth 19%). Forty-five percent of a student’s second-semester Practice course grades consisted of their outcomes on four exams (three midterms, worth 10% each, and a final exam worth 15%). Approximately 240 students attended whole-class meetings, which incorporated 150 min for lecture, 50 min for recitation, and 3 h of laboratory each week.

In the Curriculum Reform, courses were structured according to Chemistry, Life, the Universe, and Everything.^53^ In this course, 66–76% of class time and greater than 30% of assessment tasks were dedicated to predicting, explaining, or modeling phenomena.^43,45,54^ This environment also had prerequisites of enrollment requiring a “designated” pre-college math test score or the completion or concurrent enrollment in a college-level mathematics course.^55^ Sixty-five percent of a students’ grade consisted of outcomes on four tests (three midterms, worth 15% each, and one final exam, worth 20%). Approximately 340 students per semester enrolled for courses that incorporated 150 min of lecture and one 50 min recitation section per week.

### Statistical Analysis

#### Sample

Prior research identifies chemistry students scoring in the bottom quartile of pre-college math test scores as “at-risk” for unfavorable course outcomes as measured by test scores and pass rates.^25,31,32,56^ These students have been disproportionality represented by those who identify as women, Black, or Latinx.^30,34−38^ Aggregated across learning environments, the same appears valid for the current sample (see Table 1).

**Table 1:** Pre-college Math Test Scores as a Proxy for Educational Access in a Racially Unjust Society^b^

		Pre-College Math Test Score^a^		
Student Group	Sample Size	T3Qs (%)	BQ (%)	No Score (%)
Overall	4541	60	24	16
Asian	377	63	19	18
Black	238	30	55	14
First Nations	7	43	43	14
International	273	45	3	52
Latinx	223	48	35	17
Multiracial	191	63	24	13
Pacific				
Islander	4	25	25	50
Not Reported	101	80	11	9
White	3127	64	24	13

a Students scoring in the top three (T3Qs) and bottom (BQ) quartiles of pre-college math test scores relative to their cohort.

b Categories with small sample sizes are not merged to avoid erasing the experiences of minoritized identities.

Overall, 24% of students scored in the bottom quartile of math test scores. If these measures were equitable, we would expect 24% of students within each social construct (e.g., race or ethnicity) to also score in the bottom quartile. However, students who identify as Black (55%), Indigenous (43%), and Latinx (35%) were disproportionately represented among those scoring in the bottom quartile. Given the lack of evidence for biological or behavioral differences across students of differing socially constructed identities,^57,58^ we concluded that these disparities were societal, reflecting inequities in access to pre-college mathematics preparation. Thus, we hypothesized that these students would be most vulnerable to systemic changes in assessment design, especially given the emphasis on mathematics typical of college chemistry exams.

#### Coding

The final exams at either institution often repeated assessment tasks from the midterm exams; thus, we focused solely on midterms. Each course, first- and second-semester general chemistry, at both institutions (four classes in total) administered three midterms to students within the term. Overall, 352 assessment tasks (204 multiple-choice and 148 short-answer) were collected and coded as follows:

**Mechanistic Reasoning**—Assessment tasks requiring students to identify underlying (i.e., at a scalar level below) causes of phenomena (e.g., a change or observation) to make predictions or explain how the phenomenon happened. We focused attention on mechanistic reasoning rather than other sorts of thinking deemed desirable by the community (e.g., 3D learning,^6^ conceptual understanding^59^) for two main reasons: (1) an extensive body of literature in the philosophy of science, science education, and chemistry education has precisely defined what “mechanistic reasoning” means^7,8,60^ and (2) unpacking causes for observable events of interest to chemists and chemistry students requires connecting the behavior of lower scale entities (e.g., atoms and molecules) to an observable event.^61−63^
**Math**—Assessment tasks requiring multiplicative , proportional (If , increasing *C*, decreases *A*), or functional (log(*B*) = *A*) reasoning. We chose to exclude counting strategies or additive thinking from those tasks coded as “math” as they manifest in chemistry courses as balancing equations, calculating oxidation numbers, formal charge, and so forth and were common subtasks diluting the utility of this code.

Items could be described by more than one code (i.e., both mechanistic and math) or neither code. Interrater reliability between two researchers for all assessment tasks was consistently at or above moderate, with the agreement being achieved 91–93% of the time and values of Cohen’s Kappa ranging from 0.77 to 0.86. Researchers met to discuss discrepancies; coding to 100% consensus.

#### Statistical Tests

Once assessments were coded, descriptive statistics (frequencies and percentages) were used to address the first objective: identify the extent to which mechanistic reasoning is emphasized on assessments in general chemistry courses. For the second objective, we relied on binomial logistic regression to estimate the maximum likelihood of each student passing the course. This allowed us to model what percentage of correctly answered assessment tasks students (on average) would need to achieve to make passing the course most likely. This approach also allowed us to quantify how the probability of a student passing the course changed when the models were fit to the percentage of math or mechanistic assessment tasks the students answered correctly (see eq 1).
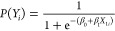
1where *P*(*Y*_*i*_) is the probability of a student *i* passing the course, e is the base of natural logarithms, β_0_ is the number of students expected to pass the course when the percentage of assessment tasks (*X*) answered correctly is 0, and β_1_ is the difference in *P*(*Y*_*i*_) for each one-unit change in the percentage of assessment tasks student *i* answered correctly (*X*_1*i*_) each additional assessment task if all other variables are held constant.

Using a threshold of >0.5 to dichotomize probabilities into “pass” or “fail”, we then calculated students’ predicted pass rates.

**Figure 1:**
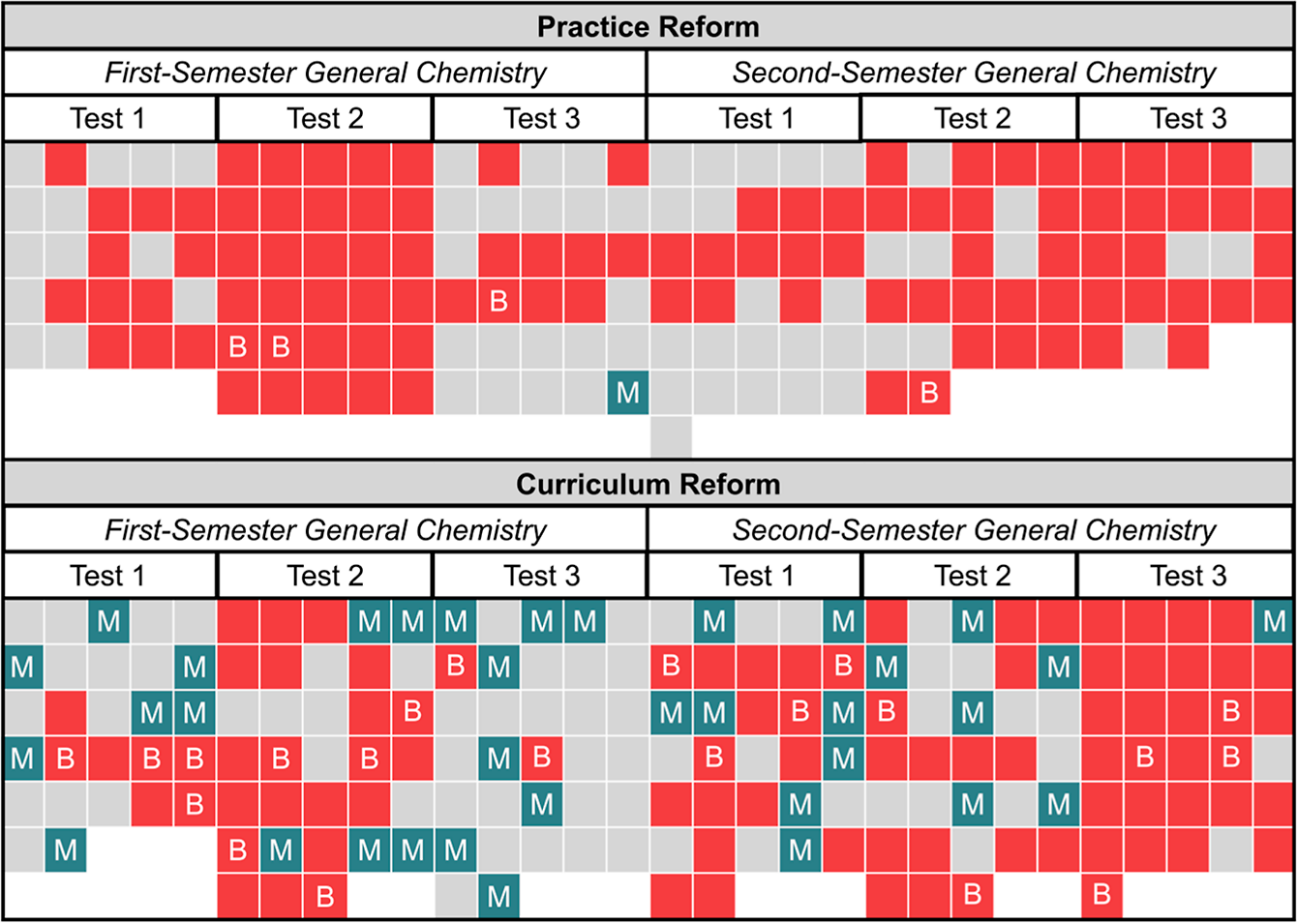
General chemistry assessments emphasized math over mechanistic reasoning. Emphases of individual assessment tasks administered in two learning environments (reformed by either instructional practice or curriculum). Red = math only; blue with an M = mechanistic reasoning; red with a B = both math and mechanistic reasoning; gray = neither math nor mechanistic reasoning.

Given the lack of mechanistic reasoning tasks in assessments administered in the Practice Reform (see Figure 1), such an analysis was feasible only using data collected from the Curriculum Reform. It was replicated across the course sequence (first- and second-semester general chemistry). All six logistic regressions (one for each of three assessment types across both courses) from data collected at the Curriculum Reform setting were statistically significant (*p* < 0.001), explained 22–60% (McFadden’s pseudo *R*^2^) of the variance in passing the course, and correctly classified 77–90% of student outcomes. All data and codes used to conduct statistical analyses in the paper are present in the Supporting Information.

## Results

In two different learning environments, assessments and student responses were collected at college-level chemistry courses offered as a two-semester sequence (first and second semester). In the first, instructors collaborated to integrate active or student-centered instructional practices into large-group class meetings (the Practice Reform). In the second, teachers developed their courses from a curriculum structured around using fundamental disciplinary ideas in chemistry (e.g., energy, electrostatic, and bonding interactions) to predict, explain, and model phenomena (the Curriculum Reform).

### Mechanistic Reasoning Was Not the Primary Emphasis of General Chemistry Exams

From our analysis, we observed that mechanistic reasoning was not the predominant emphasis on assessments administered in either learning environment. Of the 352 assessment tasks analyzed for this study, 61 (17%) were coded as having the potential to elicit mechanistic reasoning. Figure 1 outlines a holistic view of the emphasis every test placed on math and mechanistic reasoning.

Each small square represents one test question, and the colors and letters within the squares represent if the question emphasized math, mechanistic reasoning, math and mechanistic reasoning, or neither math nor mechanistic reasoning. Math was disproportionately assessed, particularly in the Practice Reform environment.

### Practice Reform

In the Practice Reform learning environment, mechanistic reasoning was rarely assessed (5 of 164 tasks, or 3%). All five mechanistic reasoning tasks were designed as short-answer questions. Four of the five required math (multiplicative, proportional, or functional mathematical reasoning as defined in the Materials and Methods). An example is provided below: **Task 1**

*0.243 g of a solid triprotic acid, H_3_A, is dissolved in water and an excess of sodium carbonate, Na_2_CO_3_, is added to the resulting acidic solution. All of the carbon dioxide released is collected yielding 52.0 mL of the gas at a temperature of 298 K and a pressure of 680 mmHg.*

*(A) How many moles of CO_2_ are collected?*

*(B) Determine the molar mass of the acid.*

*(C) If an excess of sodium sulfite (Na_2_SO_3_) was substituted in place of the sodium carbonate for the analysis, all other conditions being kept the same, identify the gas that would be collected in place of the CO_2_*.

*(D) If an excess of sodium sulfite was substituted in place of sodium carbonate for the analysis, all other conditions being kept the same, would the mass of gas collected be higher than, lower than, or the same as the mass of CO_2_ collected? Explain.*

Of the four parts of task 1 shown, only part D has the potential to elicit evidence of mechanistic reasoning. In part D, students were asked whether the difference in mass between carbon and sulfur would impact the mass of gas produced in the chemical reaction. A student could respond:

*“The mass of the gas (SO_2_) collected from the acid/base reaction would be higher when reacted with sodium sulfite than the mass of CO_2_ produced from a reaction with sodium carbonate because sulfur weighs more than carbon.”*

Because this task prompts students to link a characteristic of a lower scale entity (atomic mass, in this case) to an observable phenomenon (i.e., the mass of gas collected), we consider part D to have the potential to elicit a response in which students use mechanistic reasoning. This contrasts with parts A–C of task 1, which ask students to determine the number of moles and molar mass of the acid and identify the product of a reaction, respectively. For parts A–C, the requisite skills include aspects of mathematical reasoning, which we define as any type of proportional reasoning or algebraic manipulation. In considering task 1 as a whole, we view students’ engagement with mechanistic reasoning to be deemphasized relative to calculations, which was typical in the Practice Reform data set. Indeed, a strong emphasis (100 of 164 tasks, or 61%) was placed on assessment tasks requiring math in the Practice Reform. The remaining 59 tasks (36%) emphasized executing other skills (e.g., nomenclature, oxidation states, and electron configurations) without requiring one to connect the output of these skills to how and why phenomena happen.

#### Curriculum Reform

**Figure 2:**
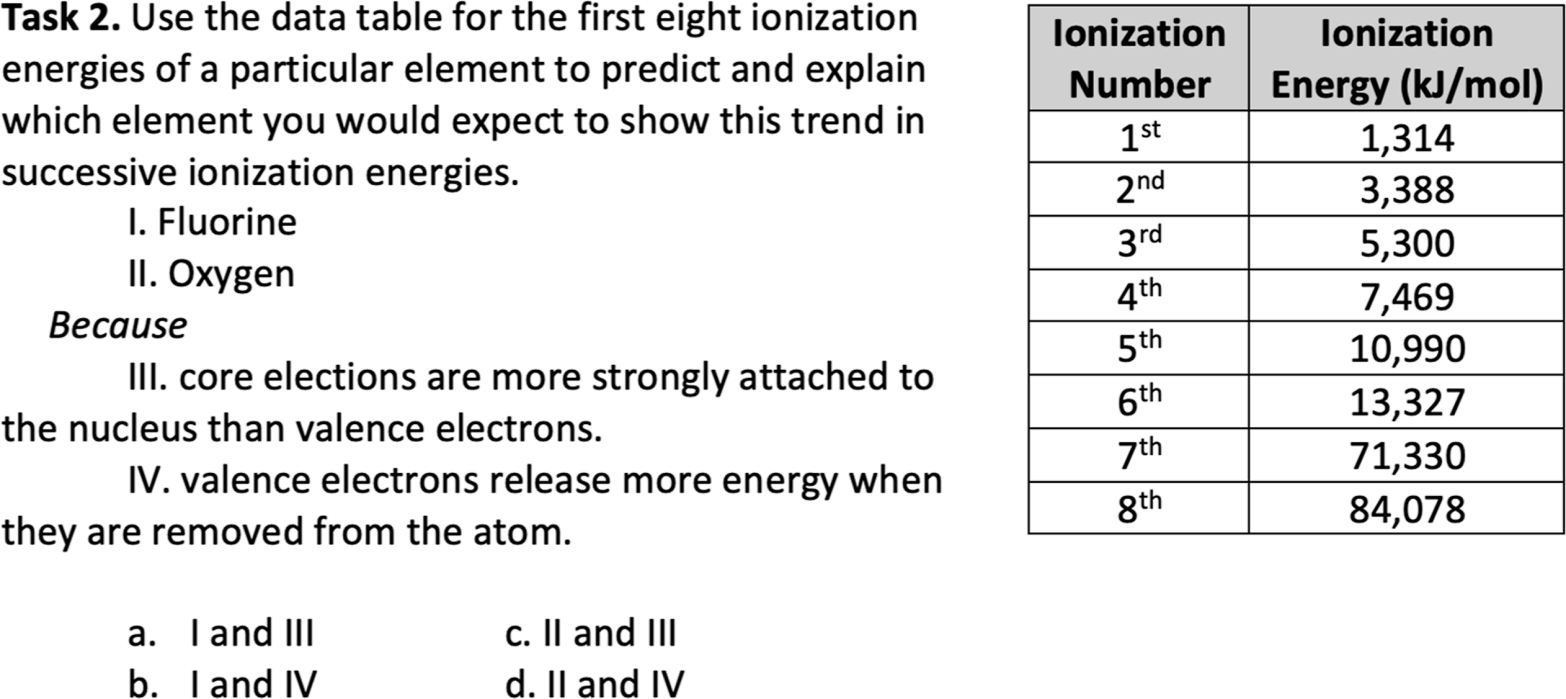
Exemplar mechanistic reasoning task. This task emphasizes mechanistic reasoning through applied proportional reasoning.

**Figure 3:**
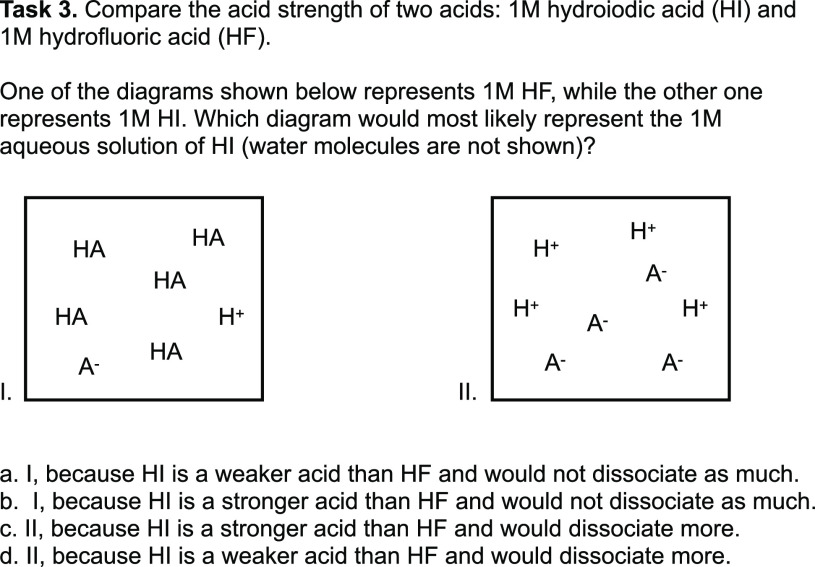
Exemplar mechanistic reasoning task. This task emphasizes mechanistic reasoning without the application of mathematics.

By comparison, the Curriculum Reform placed 10 times the emphasis on mechanistic reasoning (56 of 188 tasks, or 30%). These tasks varied in format: many (40) were multiple-choice, and less than half (21) required math. Examples of each can be found in Figures 2 and 3.

While no calculations are required to solve task 2, proportional reasoning is needed to relate ionization energy and atomic radius (e.g., the smaller the radius, the greater the ionization energy required to remove a valence electron). Task 3 was representative of mechanistic reasoning tasks assessed at the Curriculum Reform that did not require mathematics (see Figure 3).

In both tasks, students are asked to reason about the underlying causes of the chemical phenomena presented (changes in ionization energy and differences in dissociation).

Assessments administered in the Curriculum Reform placed 10 times the emphasis on mechanistic reasoning (30% of tasks) relative to the Practice Reform (3% of tasks). Yet, as in the Practice Reform, mathematical reasoning was the predominant emphasis of assessments (90 of 188 tasks, or 48%). Further, mathematical reasoning was increasingly emphasized as the course sequence progressed from the first to the second semester (see Figure 1).

In the next section, we use student responses to various types of assessments tasks to predict the impact of assessment emphasis on their probability of passing or failing first- or second-semester general chemistry in the Curriculum Reform. Data in the Practice Reform could not be used for this analysis as 3% of assessment tasks were too few to fit a statistical model.

### Assessment Emphasis Had a Considerable Impact on Which Students Passed the Course

Using binomial logistic regression, we estimated the likelihood of a student passing the course (earning a C or higher) by their performance on tasks requiring math only or mechanistic reasoning. If how we, as instructors, define academic success via the types of assessment tasks we emphasize has an impact on whether a student passes, the question becomes, “how impactful are these decisions and whom do they impact most?” Table 2 summarizes the descriptive statistics of students enrolled in first- and second-semester general chemistry courses that were part of the Curriculum Reform.

**Table 2:** Assessment Design Impacts Pass Rates^a^

Chemistry Course	Sample Size	Passed (any design)	Failed (any design)	Design Dependent	Design Dependent (%)
First Semester	2396	1889	149	358	14.9
Second Semester	957	744	30	183	19.1

a Sample sizes, the number of students who were predicted to pass or fail regardless of design, and the frequency and percentage of students whose pass rates were design-dependent.

**Figure 4:**
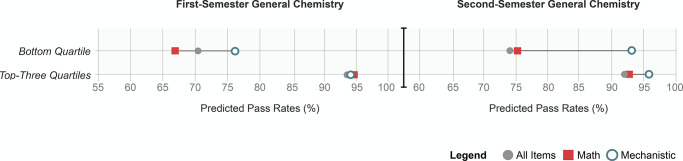
Many assessments in the general chemistry courses examined are functionally math tests and serve as barriers to students with inequitable access to pre-college mathematics preparation. Predicted pass rates for students scoring in the top three and bottom quartile of standardized math test scores as a function of their performances on all assessment tasks, math exercises, and assessments of mechanistic reasoning.

Changes in assessment emphasis were predicted to impact whether 15–20% of the student cohort passed or failed the course. Often labeled “at-risk”, the success of students scoring in the bottom quartile of standardized math test scores could be as low as 67% or as high as 93% depending on assessment design (see Figure 4).

From these data, we derived three key observations. First, pass rates were predicted to change most substantively for chemistry students scoring in the bottom quartile of standardized math test scores. Second, for students scoring in the bottom quartile, predicted pass rates based on their achievement on all assessment tasks were often closer to math exercises than assessments of mechanistic reasoning (particularly in the second semester). Third, pass rates for students scoring in the bottom quartile increased substantively when academic success was defined by their performance on assessments of mechanistic reasoning and was nearly identical to their peers scoring in the top-three quartiles of standardized math test scores in second-semester general chemistry.

**Figure 5:**
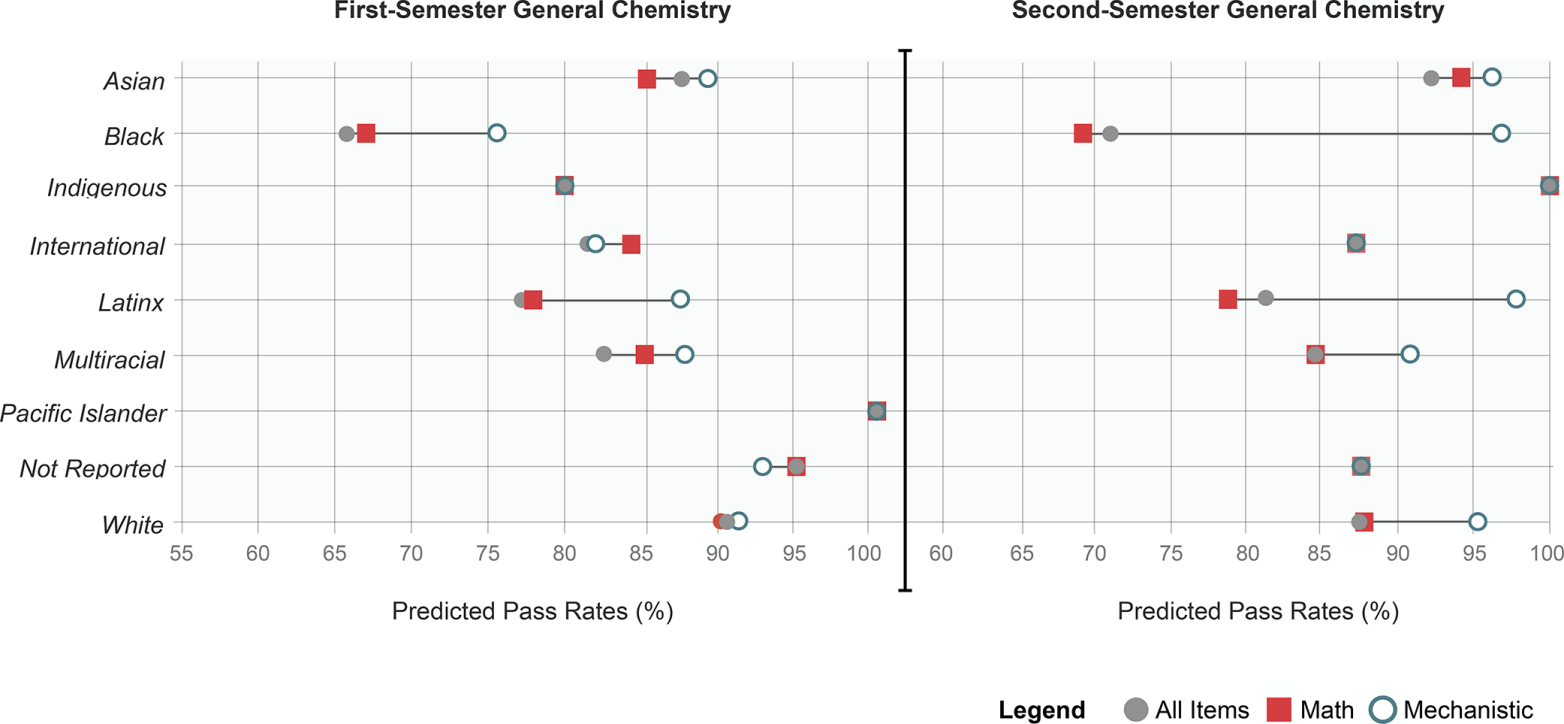
Systemic norms in assessment emphasis are predicted to have the most substantive impact on whether Black and Latinx students are selected to participate in STEM. Predicted pass rates for students of different races and ethnicities estimated by their performance on all assessment tasks, math exercises, and assessments of mechanistic reasoning.

As access to incoming mathematics preparation is not equitably accessible to students across social constructs of race and ethnicity,^30,34−38^ we disaggregated the data accordingly. We found that assessment emphasis was predicted to have the most substantive impact on Black and Latinx STEM students (see Figure 5).

Pass rates for Black and Latinx students were predicted to have the most substantive increase relative to their peers when assessments of mechanistic reasoning defined academic success. These findings were replicable and more evident in second-semester general chemistry, where math exercises were emphasized more often (revisit Figure 1). While replicable, these findings reflect predicted pass rates and not actual pass rates (see Table 3).

**Table 3:**
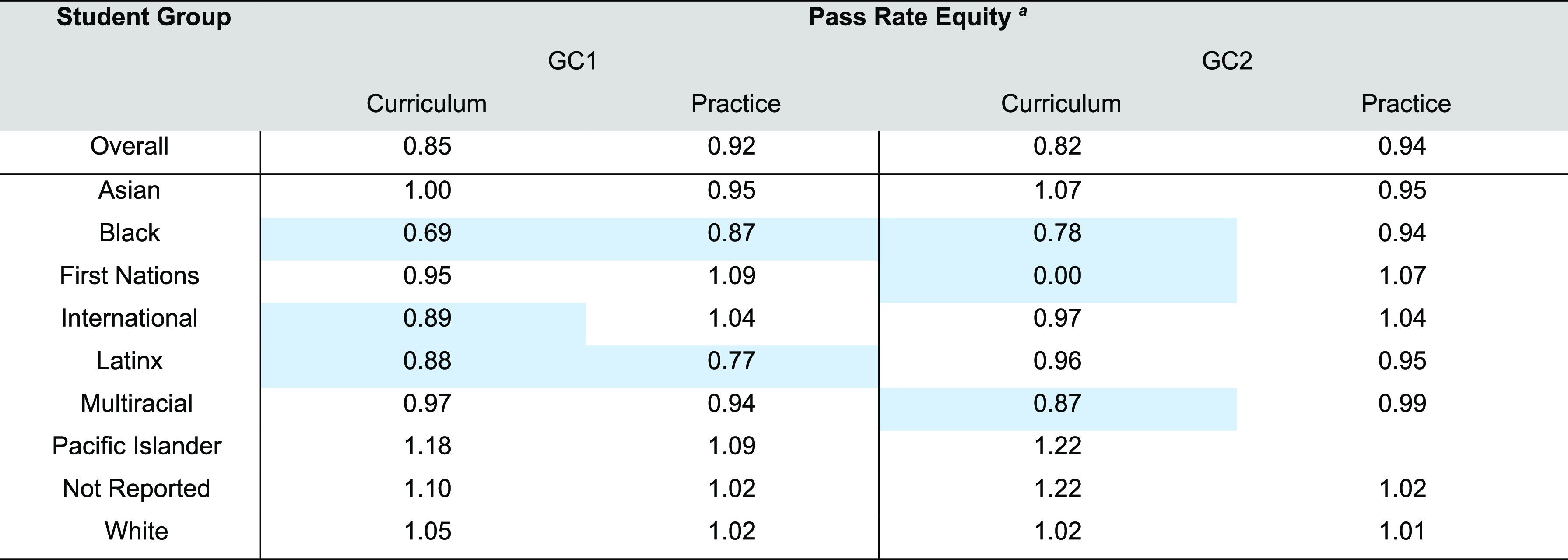
A Comparison of Pass Rate Equities Across First- (GC1) and Second- (GC2) Semester Courses Administered in the Curriculum and Practice Reform

a Pass rate equity was calculated as the proportion of students within a race or ethnicity who passed (say 0.76) divided by the overall proportion (top row) of students who passed the course (say 0.89, resulting in an 0.85). Highlighted cells indicate values less than 0.90 or populations of students who were not equitably supported in each environment. This equity index was calculated in accordance with the methods and cutoffs recommended by the Center for Urban Education.^64^

The findings in Table 3 emphasize a critical takeaway from this work: reducing the emphasis of algorithmic assessment in chemistry courses from 97 to 48% (percentage of tasks that did not assess mechanistic reasoning in Practice and Curriculum reforms, respectively) may be too insubstantial a reform to subvert systems of oppression in the educational system. The logistic regression model used in this study suggests that if students are solely assessed on mechanistic reasoning, the outcomes are statistically likely to be more equitable. We posit that increasing the proportion of assessment tasks emphasizing mechanistic reasoning alone will not be an immediate solution to educational inequality. Achieving educational equity will likely require the implementation of several systems of liberation. However, changing how we define success in science courses may help us shift the tide in science education toward experiences aligned with the practice of science and supporting all students in achieving academic success.

## Discussion

There are cohorts of chemistry students who matriculate through the general chemistry courses having little experience in reasoning about the underlying causes of phenomena. In the Practice Reform, mechanistic reasoning was rarely assessed (3%, see Figure 1). While 10 times this emphasis was observed in the Curriculum Reform, mathematics remained the predominant emphasis (48%) of the assessments administered. Reforms in how we teach offer one strategy for improving STEM courses; however, we would argue that what we are teaching, why, and whom it impacts require our attention.

### Problem

It is well known that college physical science courses, and chemistry courses in particular,^44−47^ place overwhelming emphasis on assessing skills and factual recall.^45,46^ Indeed, we previously found that 50–79% of the points awarded in high-stakes chemistry assessments required students to calculate a value without applying the outcome of that calculation to evaluate some aspect of a phenomenon.^12^ Responses to such items let us infer facility with math skills but provide no insights into whether students can link molecular behavior to how and why observable events happen. Additionally, skill- and math-heavy chemistry tests message that rote execution of algorithms is the point of the class (and possibly the discipline).

We theorize that this substantial focus on mathematical skills observed in high-stake chemistry assessments is one example of how systemic norms perpetuate exclusion. Another example can be found in efforts to predict which students are “at-risk” for unfavorable outcomes in science courses,^19,21,25−27,31,37,65−68^ often relying on students’ precollege math test scores.

The evidence generated in this study subverts the assumption that students with lower math test scores cannot succeed in STEM. While disproportionately excluded from access to pre-college mathematics preparation (Table 1), Black and Latinx students excelled in assessments of mechanistic reasoning (Figure 5). However, where emphasis on mathematic skills increased (Figure 1), educational inequity worsened. The pass rates as predicted by students’ performance on all tasks and tasks requiring math grew increasingly similar (Figures 4 and 5). We argue that students with lower math test scores are not “at-risk” of unfavorable outcomes but are put at increasing risk of exclusion from STEM careers through the overemphasis of rote mathematics in gateway science courses.

Stated succinctly, assessing mainly math skills in general chemistry misrepresents the intellectual work at the heart of the discipline while acting to exclude students marginalized by the education system. These findings may explain part of the persistent inequities observed in the retention of Black, Indigenous, and Latinx students in STEM degree programs.^30,34−38^

### Potential Solution

The results of this study indicate a potential path forward for advancing equity in science courses. By redefining academic success from students’ performance on math exercises to their engagement in mechanistic reasoning about phenomena, pass rates for students scoring in the bottom quartile of math test scores increased from 66.6 to 76% in first-semester chemistry courses (see Figure 4). This reduced equity gaps in student outcomes by 9.9%, with pass rates for Black and Latinx students predicted to increase by 22 and 14%, respectively (see Figure 5). In second-semester chemistry, where mathematics was increasingly emphasized on assessments administered in the Curriculum Reform (see Figure 1), the predicted pass rates of students scoring in the bottom quartile increased from 75 to 92.9%, nearly matching that of their peers who scored in the top-three quartiles (95.6%) and reducing the equity gap by 14.7%. The predicted increase in the pass rate for Black students enrolled in second-semester chemistry courses was substantial, improving from 69 to 96.6%. As tasks progress from calculating or executing a skill toward making predictions, reasoning about, or explaining the underlying causes of phenomena, equity was predicted to improve for these learning environments.

The noteworthy success of Black and Latinx students on mechanistic reasoning tasks merits further unpacking. We should first note that this finding is consistent with prior work by Lin and colleagues,^48^ who found that students from minoritized groups often excelled at “conceptual” but not algorithmic tasks. Although the definition of “conceptual” is fuzzy,^59^ mechanistic reasoning tasks certainly fall under this umbrella. Why might minoritized students be particularly successful at tasks requiring construction or selection of causal accounts for phenomena? Clark and Seider’s scholarship might provide some insights.^49^ Recall that they reported the role of curiosity in the unique development of Black and Latinx students’ analytical thinking, critical consciousness, and involvement in activism. Clark and Seider’s findings suggest that Black, Indigenous, and Latinx students often excel in learning environments that define academic success via engagement in nuanced reasoning (e.g., mechanistic reasoning). Accordingly, it may be that Black, Indigenous, and Latinx students are uniquely well-equipped for connecting the behavior or lower scale actors to how and why phenomena occur. The present study together with these works subverts assumptions that “at-risk” students are not as successful in science, providing further evidence of their assets in critical thinking and reasoning.

This study emphasizes how we, as educators, wield considerable power to exacerbate, perpetuate, or alleviate educational inequity in our classrooms through how we choose to define “success” in our classes. Therefore, communities of science educators are encouraged to:

(1) Question the impact of societal norms, policies, and practices on how academic success is defined and decide what and who we value for participation in STEM careers.
(2) Disaggregate measures of interest along socially constructed categories to examine the relative impact of these norms on different groups of students.
(3) Implement more focused evaluations of educational equity (e.g., outcomes on individual assessment tasks) than pass/fail rates.

For educators, we offer more detailed implications on centering curricula (and more specifically, assessments) on the intellectual work of scientists.

### For Educators: Centering Curricula on Phenomena

Instructors are encouraged to reflect on their use of learning activities and assessments to (1) ensure that mechanistic reasoning is foregrounded in assessment and other aspects of the course (e.g., lesson time and homework) and (2) regularly evaluate assessment tasks by equity for populations of interest within student groups.

**Figure 6:**
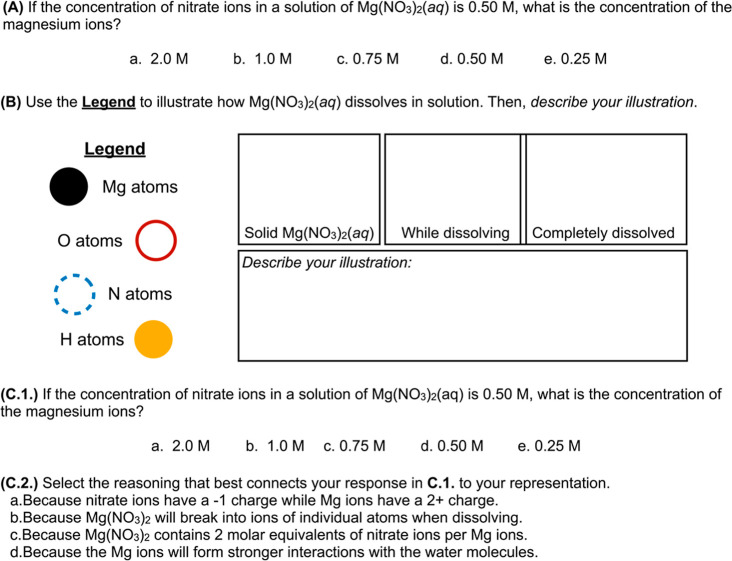
Revising chemistry assessments to deemphasize rote calculations in favor of mechanistic reasoning. (A) A multiple-choice chemistry task emphasizing rote calculation, (B) a short-answer task emphasizing mechanistic reasoning, and (C) a multiple-choice task emphasizing mechanistic reasoning.

To provide educators with a practical example of how the findings in this study could be applied in the classroom, consider the assessment tasks in Figure 6.

The items provided are similar; however, tasks B and C position student responses within the context of a phenomenon—Mg(NO_3_)_2_ dissolving in water—and ask for reasoning to explain why this phenomenon occurred.

The instructor could assign tasks B and C on an assessment in practice. If grading short-answer responses is not feasible, instructors could provide task B as a guiding tool for students and not grade their responses. Students may benefit from discussing and comparing their illustrations during or after the assessment. Instructors facilitating this discussion might emphasize that illustrations and descriptions should be productive, for and from the perspective of students, in explaining the focal phenomenon. Additional support for constructing assessment items that can elicit mechanistic reasoning may be found in several published articles.^44,69^

To evaluate the impact of assessment designs on equity, instructors could identify groups of interest and calculate the difference in means between these groups. This will allow instructors to readily identify tasks separating students, not by the knowledge they possess, but by systemic inequities imposed upon those who share a societally derived identity. We have provided a spreadsheet and a corresponding user guide in Supporting Information to aid in these efforts.

Finally, note that our findings drew on data collected in the context of a transformed curriculum that coherently emphasized mechanistic reasoning on high- and low-stake assessments and during class. Increasing the extent to which assessments emphasize mechanistic reasoning should not be perceived as a one-size-fits-all solution for instilling equitable pedagogical practices. It will not be enough to change exam questions in a course that otherwise emphasizes skills and facts. While these examples provide suggestions for revising assessment items based on the findings of this study, more work is needed to support instructors in developing equitable assessments that engage students in purposefully integrating scientific knowledge and activities to figure out phenomena. Although the transition to providing opportunities to engage in mechanistic reasoning can be challenging, we hope that educators will see these implications as an opportunity to support better their students’ goals and desires for engaging in science in science courses.

## Limitations

It is important to note that data upon which our findings are based were collected from a transformed curricular context in which mechanistic reasoning was relatively more emphasized in class, homework, and exams than may be observed in other courses.^12,45,50^ We cannot generalize our findings beyond this transformed context. Similarly, we recognize that this study was performed for 1 year of a general chemistry course and there are chances that the results would differ across several years. However, our results support the extant literature on the relationship between math preparation and success in chemistry, and we encourage practitioners to use the tools provided to reflect on their own practices, keeping in mind general trends from this study and current literature. Additionally, student responses to exam prompts enable limited inferences about the ideas students activated in each context on a particular day. We make no claims about durable “understandings” or “misunderstandings” from our data corpus. Students who did not select the “correct” answer to a given question may have had more success with a differently worded prompt. Finally, student grade predictions were based entirely on their responses to multiple-choice assessment tasks. These findings may not be generalizable at institutions where short-answer questions dominate summative assessment practices.

In terms of equity, predictions imply that these reforms would benefit equity in a transformed curricular context. However, the actual pass rates listed in Table 3 highlight the voices of scholars advocating for equity-centered reforms in science education rather than curricular (or pedagogical reforms) that evaluate equity post hoc.^70−73^ Findings in Table 3 also suggest that curricula weighting rote calculations and mechanistic reasoning equally may still be considered inequitable in course outcomes. This research could be expanded in a future study by comparing the equity emerging from different learning environments with assessments solely centered on mechanistic reasoning and comparatively have little emphasis on rote calculations. Alternatively, researchers could compare various approaches to assessment design, generating evidence that could inform equity-centered assessment designs aligned with the application of mechanistic reasoning in chemistry. Finally, in this study, “academic success” was equated to students’ pass or fail rates. While a potentially normative argument, it fails to acknowledge how students (particularly marginalized students) describe and measure their academic success.^74^

Another critical limitation of the work worth addressing is the labels used to categorize students by socially constructed identities of race or ethnicity. The categorical data collected for this study was subject to regulation by the United States federal government.^75^ These federal regulations require people to self-identify as a single category of gender (despite the fallacy of binary gender) and conflate their ethnic-racial identities by selecting only one (e.g., students were unable to identify as Black and Latinx).

Acknowledging that all data cannot “speak for itself” and instead reflect the hegemonic (or dominating) power structures that created them,^76,77^ we attempted to rectify some of the terminology used to identify student groups in a manner consistent with efforts in justice-oriented research specifically as they pertain to race and ethnicity.^78−80^ These efforts included changing: (1) the gendered term “Hispanic or Latino” to a more inclusive “Hispanic or Latinx”; (2) the inaccurate, colonizing term of “American Indian or Alaska Native” to a respectful category acknowledging Indigeneity “First Nations”; and (3) the dehumanizing term “non-resident aliens” to the more geographically accurate term “International”. Unfortunately, we could not separate race and ethnicity as the original categories did not differentiate (e.g., these selections did not allow a student who identifies as Black to also identify as Hispanic or Latinx). We support further efforts to reform federal regulations and encourage researchers to allow students more input or open-ended methods of self-identification. While changing these labels can misrepresent a student’s social identity, we recognize the harm in perpetuating such terminology. We encourage readers to reach out to any of the corresponding authors to discuss this issue further.

## Supporting Information Available

User guide to support educators in enacting by-item analyses of equity (XLSX)
Reproducible R markdown including data and analysis for statistical analyses conducted in this study (ZIP).
